# Muscle architecture and passive lengthening properties of the gastrocnemius medialis and Achilles tendon in children who idiopathically toe‐walk

**DOI:** 10.1111/joa.13464

**Published:** 2021-06-09

**Authors:** Carla Harkness‐Armstrong, Constantinos Maganaris, Roger Walton, David M. Wright, Alfie Bass, Vasilios Baltzopoulos, Thomas D. O’Brien

**Affiliations:** ^1^ Research Institute for Sport and Exercise Sciences Liverpool John Moores University Liverpool UK; ^2^ Alder Hey Children’s NHS Foundation Trust Liverpool UK

**Keywords:** Achilles tendon, equinus, idiopathic toe‐walking, muscle architecture, ultrasound

## Abstract

Children who idiopathically toe‐walk (ITW) habitually operate at greater plantarflexion angles and thus, at shorter muscle‐tendon unit (MTU) lengths than typically developing (TD) children. Therefore, it is often assumed that habitual use of the gastrocnemius muscle in this way will cause remodelling of the muscle‐tendon architecture compared to TD children. However, the gastrocnemius muscle architecture of children who ITW has never been measured. It is essential that we gain a better understanding of these muscle‐tendon properties, to ensure that appropriate clinical interventions can be provided for these children. Five children who ITW (age 8 ± 2 years) and 14 TD children (age 10 ± 2 years) participated in this study. Ultrasound was combined with isokinetic dynamometry and surface electromyography, to measure muscle architecture at common positions and passive lengthening properties of the gastrocnemius muscle and tendon across full range of motion. Regardless of which common condition groups were compared under, both the absolute and normalised to MTU muscle belly and fascicle lengths were always longer, and the Achilles tendon length was always shorter in children who ITW than TD children (*p* < 0.05; large effect sizes). The passive lengthening properties of the muscle and tendon were not different between groups (*p* > 0.05); however, passive joint stiffness was greater in children who ITW at maximum dorsiflexion (*p* = 0.001) and at a joint moment common to all participants (*p* = 0.029). Consequently, the findings of this pilot study indicate a remodelling of the relative MTU that does not support the concept that children who ITW commonly experience muscle shortening. Therefore, greater consideration of the muscle and tendon properties are required when prescribing clinical interventions that aim to lengthen the MTU, and treatments may be better targeted at the Achilles tendon in children who ITW.

## Graphical abstract

We used ultrasound to measure the gastrocnemius medialis muscle architecture in children who idiopathically toe‐walk (ITW). Despite habitually operating at shorter muscle‐tendon unit lengths, children who ITW presented with longer muscle and shorter tendon lengths than typically developing children. Therefore, these findings indicate a remodelling of the relative muscle‐tendon unit that does not support the concept that children who ITW commonly experience muscle shortening.

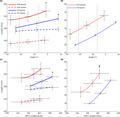

## 1 INTRODUCTION

Children who idiopathically toe‐walk (ITW) ambulate with the absence of a heel contact with the floor at initial contact, despite no known orthopaedic or neurological cause (Pomarino et al., 2016). Persistent, untreated toe‐walking can lead to equinus contracture (Solan et al., 2010), therefore clinical treatments are often prescribed to lengthen the gastrocnemius muscle‐tendon unit (MTU). However, the alterations in muscle and tendon properties that lead to this reduced range of motion (ROM) of children who ITW have never been measured. Understanding these muscle‐tendon properties may improve our understanding of the pathology and better target clinical interventions for these children.

In typically developed gait, the movement kinematics and muscle architecture combine so that the gastrocnemius medialis muscle operates close to optimal length, which favours the economical production of high contractile force (Fukunaga et al., 2001). This association between structure and function is common (Arnold & Delp, 2011) because muscles remodel in length according to the used ROM (Matano et al., 1994; Williams & Goldspink, 1973). Children who ITW ambulate with a greater plantarflexion angle compared with typically developing (TD) children and therefore, are likely operating at shorter gastrocnemius muscle and whole MTU lengths. Thus, it is expected that habitual use in this way will cause remodelling of the gastrocnemius medialis muscle architecture compared to TD children.

Equinus also presents in many children with cerebral palsy (CP). Although the pathway of cause‐and‐effect is more complex in CP (Gage & Novacheck, 2001), it is well documented that children with CP have shorter gastrocnemius MTU lengths (Kruse et al., 2018), which are, in turn, composed of shorter (Barrett & Lichtwark, 2010; Fry et al., 2004) and stiffer (Willerslev‐Olsen et al., 2018) medial gastrocnemius muscle bellies, shorter fascicle lengths (Kalkman et al., 2018; Matthiasdottir et al., 2014; Mohagheghi et al., 2008), and longer Achilles tendon lengths (Barber et al., 2012; Kalkman et al., 2018; Wren et al., 2010) than TD children. The passive lengthening properties of the muscle and tendon are also altered in children with CP (Kalkman et al., 2018). Thus, to treat these muscle‐tendon alterations, and to prevent permanent remodelling of the muscle to these shorter, sub‐optimal lengths (contracture), clinical interventions (e.g. serial casting, botulinum toxin‐A injections, surgery) are prescribed to restore dorsiflexion ROM and a typical heel‐toe gait pattern, by lengthening the gastrocnemius MTU (Alhusaini et al., 2011; Brouwer et al., 2000; Jahn et al., 2009; Kay et al., 2004).

As children who ITW ambulate with a similar locomotor dysfunction to many children with CP, it is often assumed that they will have undergone similar muscle and tendon alterations within the gastrocnemius MTU complex. The expectation of shorter muscle belly and MTU lengths would explain why children who ITW are often prescribed with similar interventions as their CP counterparts (Williams et al., 2016). However, the muscle and tendon properties have never been measured in children who ITW. Therefore, it is not known if the MTU length, or indeed the relative lengths of muscle and tendon differ from typical. Nonetheless, children who ITW often undergo invasive clinical interventions to lengthen the MTU. However, it is not known what the optimal procedure should be (e.g. whether the muscle or tendon should be lengthened), or the implications that such interventions will pose. Consequently, if the assumptions of muscle length, and therefore function, are in any way incorrect, this may explain why the current medium to long‐term effectiveness of interventions are often poor for children who ITW (Dietz & Khunsree, 2012; van Kuijk et al., 2014).

Although it is rather rare for equinus to persist beyond early childhood in children who ITW (Engström & Tedroff, 2012), the gait pathology does continue in a small number of children who often require clinical treatments. Whilst this makes children who ITW a difficult population to recruit experimentally, it is essential that we gain a better understanding of the muscle and tendon architectural properties in these children. This will help to better understand the pathology and better inform current clinical practice for these children. Thus, the aim of this study was to measure the architectural structure and passive lengthening properties of the gastrocnemius medialis muscle and Achilles tendon in children who ITW and TD children across the ROM. We hypothesised that children who ITW would exhibit remodelled gastrocnemius MTU lengths, which would be composed of a shorter muscle belly and a longer Achilles tendon length than TD children.

## 2 METHOD

### 2.1 Participants

Five children who bilaterally ITW (male *n* = 2; female *n* = 3; age 8 ± 2 years; height 1.38 ± 0.15 m; body mass 45.2 ± 26.7 kg) and 14 TD children (male *n* = 5; female *n* = 9; age 10 ± 2 years; height 1.39 ± 0.11 m; body mass 37.8 ± 17.5 kg) participated in this study. Children who ITW were recruited from outpatient lists at a hospital gait laboratory and orthopaedic clinics. All children had a confirmed diagnosis of idiopathic toe‐walking based on an exclusion of all other diagnoses. Children who ITW had not undergone any orthopaedic intervention (surgical or casting) within 2 years prior to the study and had not been given botulinum toxin‐A injections within 6 months prior to the study. Two children who ITW had received carbon fibre insoles and splints 3 years prior to participation. The remaining three children who ITW had significant fixed equinus contracture (Range: −12 to −30°) and had received no orthopaedic intervention. All TD children were free from neuromuscular and skeletal disorders and were free from lower limb injuries for 6 months prior to the study. This study was completed in accordance with the recommendations of both the institutional and National Health Service (UK) ethics committees (18/NW/0526). Written informed consent was obtained from parent/guardians, and written assent was given by children, in accordance with the declaration of Helsinki.

### 2.2 Selection of outcome measures

The lengths of the medial gastrocnemius muscle belly, fascicles and tendon were compared between groups as absolute lengths and relative to total MTU length. To ensure that comparisons were not confounded by variations in relative ROM or passive tension amongst children, between‐group comparisons were made at a joint angle (−15°; corresponded to the average 0 Nm joint moment of TD children and was close to the end ROM common to all participants) and MTU length (365 mm) common to all participants, and at individual 0 Nm joint moment (assumed to approximate zero passive MTU force). Additionally, fascicle pennation angle and muscle thickness were compared at 0 Nm to ensure no tissue deformation (Dick & Wakeling, 2017).

However, the joint angles to achieve these specific joint positions differ between individual children and were not possible to predict a priori. Thus, physically measured data were obtained at five relative joint angles across ROM, including maximum dorsiflexion, maximum plantarflexion, and at 25% intervals between (full procedure described below). Data were then interpolated using a second‐order polynomial (*R* ^2^ range: 0.90–0.99) to calculate and compare the specific parameters at the common joint angle, common MTU length and at individual 0 Nm joint moment.

### 2.3 Measurement protocol

Data were collected in one testing session at a university laboratory. Measurements were obtained from the right leg of TD children, and the most affected leg of children who ITW, defined as the observed degree of plantarflexion angle during gait.

**FIGURE 1:**
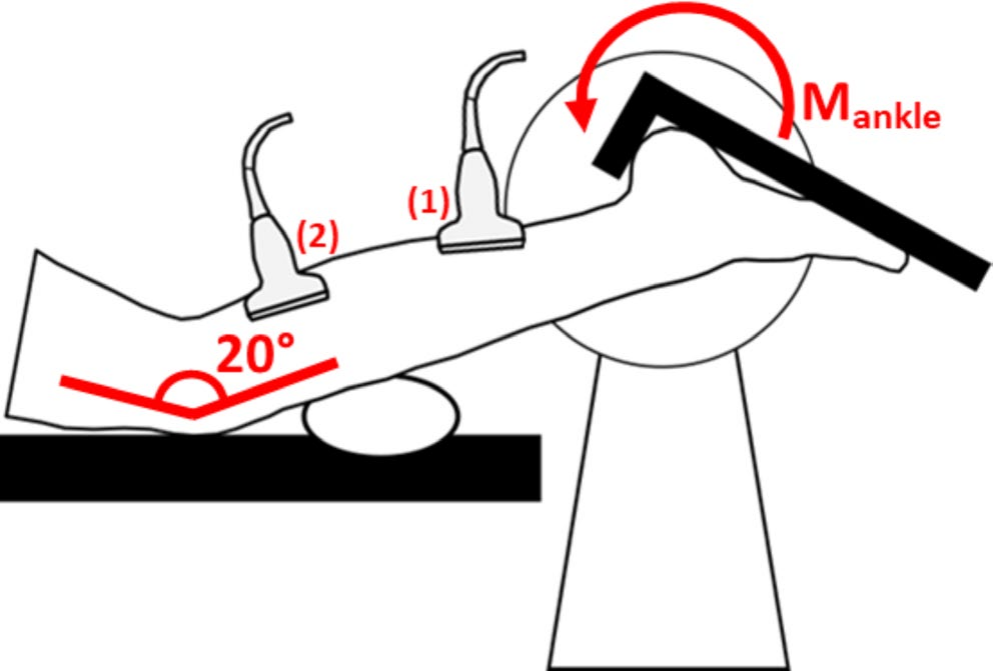
Experimental set‐up to measure gastrocnemius muscle architecture and passive ankle moment throughout range of motion on an isokinetic dynamometer. Participants lay prone, with their hip extended fully and lower limb supported so that the knee was 20° flexed. In each joint position, an ultrasound probe was used to (1) identify the myotendinous junction of the medial gastrocnemius and Achilles tendon and (2) to image the fascicle architecture from the mid‐belly of the gastrocnemius medialis

Participants lay prone on an isokinetic dynamometer (Humac Norm CSMI) bed with their hip in full extension and lower leg supported so that the knee was 20° flexed (Figure 1). The axis of rotation of the dynamometer arm was aligned with the lateral malleolus throughout passive rotation before the participant's foot was securely fastened to the footplate. A custom‐made arch support ensured that heel‐contact was maintained with the footplate across full ROM. Ankle ROM in both directions was determined by manually rotating the footplate until either (1) no further joint rotation was achieved with the application of increased joint moment or (2) the participant indicated their stretch threshold. Ankle angle and moment were sampled from the dynamometer analogue output at 1600 Hz in Acknowledge software (Biopac Systems). Net plantarflexion moment was corrected for the moment caused by the weight of the footplate. The moment of the foot was considered negligible.

To ensure the muscle was at rest for all measurements, disposable surface electromyography (EMG) electrodes (BioNomadix, Biopac Systems) were placed on the mid‐portion of the tibialis anterior and the gastrocnemius lateralis. Muscle activity was recorded synchronously with angle and moment at 1600 Hz in Acknowledge. EMG signals were inspected visually and any trials showing muscle activation were discarded.

**FIGURE 2:**
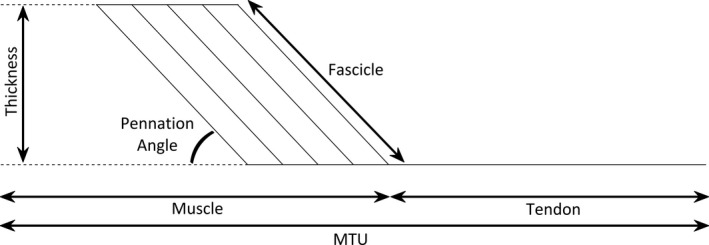
Schematic representation of the muscle and tendon architectural properties measured at maximum dorsiflexion, maximum plantarflexion, and at 25% intervals between. Muscle thickness and pennation angle were only reported at individual 0 Nm

To measure muscle, tendon and MTU length (Figure 2), the most superficial point on the posterior medial femoral condyle (assumed muscle origin) and the distal insertion of the Achilles tendon onto the calcaneus were identified using a linear B‐mode ultrasound transducer (Phillips EPIQ7) and marked on the skin with surgical marker. At each of the physically measured joint positions (maximum dorsiflexion, plantarflexion and the 25% intervals), the medial gastrocnemius myotendinous junction (MTJ) was identified with ultrasound in the sagittal plane and marked on the skin with surgical marker. Muscle and tendon lengths at each position were measured with a segmometer as the straight‐line distance between the medial femoral condyle and the MTJ, and between the MTJ and calcaneal insertion, respectively. MTU length was calculated as the sum of the respective muscle and tendon lengths, to allow for a non‐straight MTU path.

To measure fascicle length, muscle thickness and pennation angle at each joint position (Figure 2), the ultrasound transducer was moved from the MTJ to be positioned over the mid‐belly of the gastrocnemius medialis muscle so that whole fascicle length between the superficial and deep aponeuroses of the muscle could be visualised within the scan's viewing window. Although measured across full ROM, pennation angle and muscle thickness (Figure 2) were only reported at 0 Nm, as any passive force in the MTU will cause these structures to deform (Dick & Wakeling, 2017) and thus, lose physiological meaning. All ultrasound data were analysed manually in ImageJ software (ImageJ 1.51j8). All measurements of muscle and tendon architecture were averaged between two measurements at each joint position.

Passive joint stiffness was calculated for each participant by fitting a second‐order polynomial (*R* ^2^ range: 0.96–0.99) to the measured joint moment‐angle data. Similarly, fascicle pseudo‐stiffness was calculated from a second‐order polynomial (*R* ^2^ range: 0.86–0.99) fitted to the measured joint moment‐fascicle length data. For both measures of stiffness, the respective equations for each participant were then differentiated to calculate passive stiffness at maximum dorsiflexion, where passive moment is greatest, and at a passive joint moment common to all participants (0.02 Nm·kg^−1^), determined by the smallest maximum passive moment (Waugh et al., 2012).

### 2.4 Statistical analysis

All statistical analyses were completed in RStudio (RStudio 1.3.959). All variables were checked for normal distribution using the Shapiro–Wilk test and visual inspection of the q‐q plots. Student's *t* tests (normally distributed variables) and Mann–Whitney *U* tests (non‐normally distributed variables) were used to compare absolute and relative to MTU length differences between groups at a common ankle angle, MTU length and at 0 Nm joint moment, and the passive joint and fascicle stiffness’ at maximum dorsiflexion and common passive moment. Lengthening data across the full and common ROM were compared between groups using Kruskal–Wallis tests with follow‐up post hoc tests. Significance was set at *p* < 0.05. Effect sizes (ESs) were calculated using Hedge's g and considered small (>0.2), moderate (>0.5) or large (>0.8). ESs were considered unclear if the 90% confidence intervals included both substantial positive and negative values (≥±0.20) (Hopkins et al., 2009). Data are presented as means ± standard deviation (SD), unless stated otherwise.

## 3 RESULTS

**FIGURE 3:**
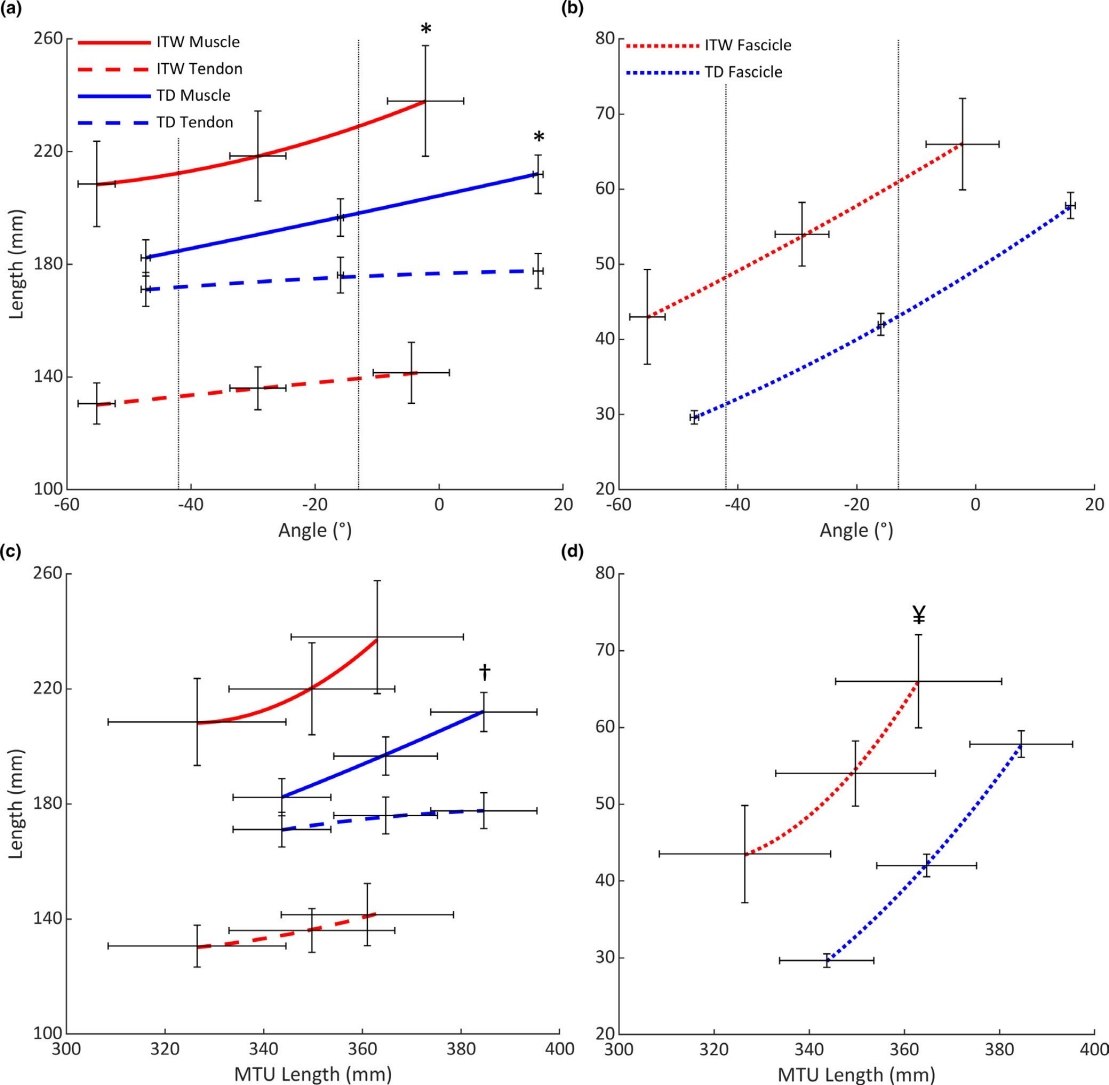
(a) Muscle/tendon length and (b) fascicle length versus ankle angle over the full range of motion. Grey dashed lines indicate the range of motion common to all participants. (c) Muscle/tendon length and (d) fascicle length versus muscle‐tendon unit length over the full range of motion. For clarity, standard error of the mean bars are shown at three joint positions only (maximum plantarflexion, 50% of range, maximum dorsiflexion). Abbreviations: ITW, children who idiopathically toe‐walk; MTU, muscle‐tendon unit; TD, typically developing children. *Significant difference between muscle and tendon lengthening throughout range of motion (*p* < 0.05). ^†^Significant difference between groups in the ratio of muscle to tendon length when plotted against muscle‐tendon unit length (*p* < 0.05). ^¥^Significant difference between groups in fascicle length when plotted against muscle‐tendon unit (*p* < 0.05)

Across the full ROM, children who ITW had longer muscle and fascicle lengths and a shorter tendon length than TD children (Figure 3). These differences were statistically significant when groups were compared at a common joint angle (−15°) (large ESs: 1.03–2.00), common MTU length (365 mm) (large ESs: 1.41–3.23) and at individual 0 Nm (large ESs: 0.94–1.68) (Table 1), despite children who ITW being significantly more plantarflexed than TD children (*p* = 0.016).

**TABLE 1:** Mean ± SD of medial gastrocnemius medialis muscle‐tendon architectural properties of children who ITW and TD children at a common joint angle (−15°), common joint moment (0 Nm) and common MTU length (365 mm)

	Absolute lengths			Percentage of MTU (%)	
	ITW	TD	ES (Hedge's g)	ITW	TD
At common angle (−15°)					
MTU (mm)	364 ± 42	373 ± 39	−0.20 ± 0.94	—	—
Muscle (mm)	229 ± 43	197 ± 25	1.03 ± 0.99	62 ± 6	53 ± 4*
Tendon (mm)	136 ± 14	176 ± 23*	−1.75 ± 1.06	37 ± 5	47 ± 4*
Fascicle (mm)	62 ± 17	42 ± 5*	2.00 ± 1.02	17 ± 3	11 ± 1*
At common moment (0 Nm)					
Ankle angle (°)	−22 ± 4	−16 ± 2*	−2.29 ± 1.06	—	—
MTU (mm)	353 ± 40	365 ± 39	−0.29 ± 0.95	—	—
Muscle (mm)	225 ± 40	197 ± 25	0.94 ± 0.98	62 ± 6	53 ± 4*
Tendon (mm)	137 ± 17	176 ± 24*	−1.64 ± 1.05	38 ± 5	47 ± 4*
Fascicle (mm)	59 ± 17	42 ± 5*	1.68 ± 0.98	17 ± 4	11 ± 1*
Thickness (mm)	17 ± 5	14 ± 3	0.73 ± 0.89	—	—
Pennation (°)	19 ± 2	20 ± 2	−0.58 ± 0.88	—	—
At common MTU length (365 mm)					
Ankle angle (°)	−8 ± 13	−32 ± 2*	3.38 ± 1.26	—	—
Muscle (mm)	231 ± 39	189 ± 24*	1.42 ± 1.02	63 ± 5	51 ± 5*
Tendon (mm)	140 ± 22	173 ± 22*	−1.41 ± 1.02	37 ± 7	48 ± 6*
Fascicle (mm)	63 ± 15	35 ± 5*	3.23 ± 1.23	18 ± 2	10 ± 4*

Abbreviations: ES, effect size; ITW, children who idiopathic toe‐walk; MTU, muscle‐tendon unit; TD, typically developing children.

* Significant difference between children who idiopathically toe‐walk and typically developing children (*p* < 0.05).

When normalised to MTU length, the muscle remained significantly longer and the tendon significantly shorter in children who ITW at a common joint angle (*p* = 0.032; large ES: 2.13), MTU length (*p* = 0.035; large ES: 1.69) and at individual 0 Nm (*p* = 0.049; large ES: 1.98) than TD children. Fascicle length normalised to MTU length was also significantly longer in children who ITW than TD children in all considered joint positions (*p* < 0.05; large ESs: 2.16–3.27) (Table 1).

At 0 Nm, MTU length was not significantly different between groups, but children who ITW had a greater muscle thickness (17 ± 5 vs. 14 ± 3 mm; *p* = 0.071; moderate ES: 0.73) than TD children. There was no difference in pennation angle between groups (19 ± 2 vs. 20 ± 2°; *p* = 0.233; unclear ES: −0.58).

Children who ITW had a significantly smaller ROM than TD children (53 ± 7 vs. 63 ± 4°; *p* = 0.001). Excluding individual maximum plantarflexion (*p* = 0.065), children who ITW were significantly more plantarflexed at all remaining physically measured joint positions than TD children (*p* < 0.05). There was no difference in muscle or tendon lengthening between groups over full ROM or a ROM common to all participants (−42 to −13°; *p* > 0.05; Figure 3a). However, muscle lengthening was significantly greater than tendon lengthening in both groups (*p* < 0.05; Figure 3a). Over the full ROM, fascicle lengthening was significantly less in children who ITW (*p* = 0.032); however, there was no difference in fascicle lengthening between groups over a common ROM (Figure 3b).

**FIGURE 4:**
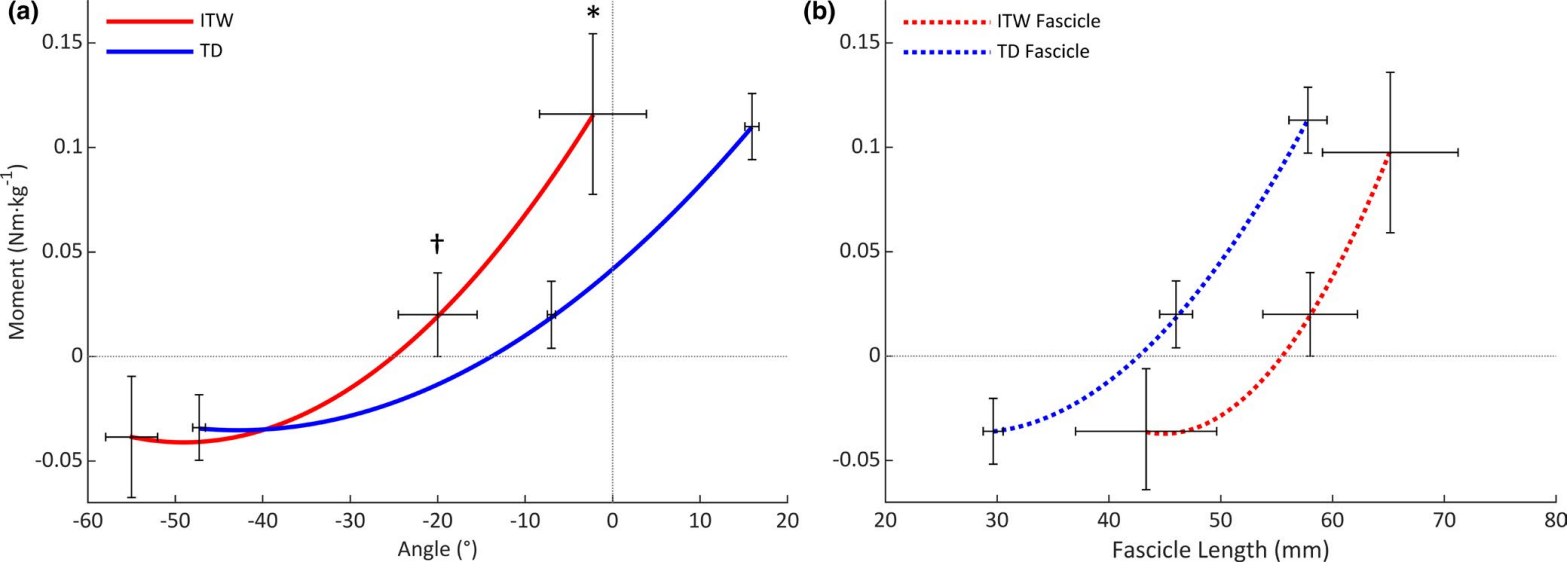
Passive ankle moment versus (a) ankle joint angle and (b) resting fascicle length across full range of motion. For clarity, standard error of the mean bars are shown at three joint positions only (maximum plantarflexion, maximum dorsiflexion and at the common joint moment that groups were compared). Abbreviations: ITW, children who idiopathically toe‐walk; TD, typically developing children. *Significant difference between groups at maximum dorsiflexion (*p* < 0.05). ^†^Significant difference between groups at common passive moment of 0.02 Nm·kg^−1^ (*p* < 0.05)

At individual maximum dorsiflexion, children who ITW had a significantly greater passive joint stiffness than TD children (0.009 ± 0.003 vs. 0.005 ± 0.002 Nm·kg·deg^−1^; *p* = 0.001; Figure 4a); however, there was no difference in the passive fascicle stiffness between groups (0.017 ± 0.011 vs. 0.010 ± 0.004 Nm·kg·mm^−1^; *p* = 0.202; Figure 4b). At a common joint moment of 0.02 Nm·kg^−1^, children who ITW also had a significantly greater passive joint stiffness than TD children (0.007 ± 0.002 vs. 0.003 ± 0.001 Nm·kg·deg^−1^; *p* = 0.029; Figure 4a); however, there was no difference in the passive fascicle stiffness between groups (0.013 ± 0.007 vs. 0.007 ± 0.002 Nm·kg·mm^−1^; *p* = 0.112; Figure 4b).

## 4 DISCUSSION

This pilot study is the first to measure the muscle and tendon architecture and passive lengthening properties of the gastrocnemius medialis MTU in children who ITW. Contrary to our hypothesis, in all considered joint positions, children who ITW had longer absolute and normalised gastrocnemius medialis muscle belly and fascicle lengths, and a shorter Achilles tendon length than TD children, despite habitually operating at shorter MTU lengths. Consequently, these findings indicate a remodelling of relative MTU lengths, which does not support the concept that these children commonly experience muscle shortening caused by a reduced ROM, as children with CP do. Therefore, clinical interventions may be better targeted at the Achilles tendon when aiming to restore MTU length and ROM in children who ITW.

To ensure that comparisons of the muscle architectural properties were not confounded by the differences in the relative ROM and passive stiffness of children, between‐group comparisons were made at a common joint angle and MTU length, and at 0 Nm joint moment. Regardless of which common condition groups were compared under, both the absolute and normalised to MTU muscle belly and fascicle lengths were always longer, and the Achilles tendon length was always shorter in children who ITW than TD children (Table 1). This was particularly surprising at 0 Nm joint moment because children who ITW were significantly more plantarflexed, but the muscle belly and fascicle lengths remained longer, and the tendon shorter, than TD children. At 0 Nm joint moment, the muscle thickness of children who ITW was greater than TD children (moderate ES: 073). This may suggest that children who ITW would be able to produce more plantarflexor force than TD children, despite the notion that plantarflexor weakness contributes to equinus gait (Hampton et al., 2003). Therefore, this should be explored in the future. Nonetheless, data from the present study cause us to reject our hypothesis that children who ITW would have shorter absolute and normalised to MTU muscle and fascicle lengths, and a longer tendon length than TD children.

As the fascicle pseudo‐stiffness was similar between groups, it appears that the reduction in ROM of children who ITW may be attributed to a shorter Achilles tendon length, which contributes to a greater ankle joint stiffness. Indeed, a shorter, which typically corresponds to a stiffer, Achilles tendon would increase the stretching stimulus experienced by the in‐series muscle and may therefore explain why the gastrocnemius muscle and fascicle lengths of children who ITW have remodelled to longer lengths than TD children (Herbert & Crosbie, 1997). The function of a shorter and stiffer tendon may allow children who ITW to better control the position of the ankle joint during gait (Alexander, 2002) and maintain plantarflexion during loading of stance without the requirement of greater muscle shortening. However, this hypothesis is also dependent on other musculoskeletal interactions and should be tested.

Treatments to increase MTU length are often based on the assumption that children who ITW will have developed similar gastrocnemius MTU alterations to those with CP (Alhusaini et al., 2011; Brouwer et al., 2000; Jahn et al., 2009; Kay et al., 2004; Williams et al., 2016). However, we have shown that the MTU length of children who ITW is composed of a greater muscle to tendon ratio than typical. Therefore, the remodelling of muscle‐tendon lengths of children who ITW are the exact opposite of that in children with CP, despite most of our patients exhibiting significant fixed equinus contracture. Consequently, although commonly associated, CP and idiopathic toe‐walking appear to be two completely different conditions and therefore, may require different treatment approaches. Data from the current study suggest that clinical interventions for children who ITW should aim to lengthen the MTU, by lengthening the muscle aponeurosis (zone 2), or increasing the length and reducing the stiffness of the Achilles tendon (zone 3) rather than lengthening the gastrocnemius muscle (zone 1). Possible conservative treatments to lengthen the tendon in isolation without the need for surgery should also be explored.

In the present study, the muscle of children who ITW lengthened significantly more than the tendon to achieve full ROM, which suggests that there is a greater relative stiffness of tendon to muscle. Therefore, during conservative interventions which aim to stretch the MTU (stretching, serial casting etc.), the more compliant structure, in this case the muscle, will receive a higher physiological stimulus than the stiffer tendon (Kalkman et al., 2019). Consequently, it is more likely that the muscle will be the structure to ‘see’ the stretch and increase in length. We have shown that the muscle length is already longer than typical in children who ITW, therefore it is possible that conservative clinical interventions could be lengthening an already long muscle. However, it is also possible that the between‐group differences in muscle length may be due to general physiotherapy and stretching interventions provided to the children who ITW in the years since diagnosis. Nevertheless, further increasing the length of the gastrocnemius muscle would cause the discrepancy between the relative stiffness of the muscle and tendon to be even greater, which may have implications for physical function in these children. However, further work is required to assess the force producing capabilities at these altered lengths and thus, the functional operating lengths of the gastrocnemius muscle in children who ITW.

Some limitations should be acknowledged. Firstly, the origin of the gastrocnemius medialis muscle was defined as the most superficial point on the posterior aspect of the medial femoral condyle. Therefore, we may have neglected differences in small portions of proximal tendon or muscle in our measurements of muscle and MTU length. However, this method is an established approach for these measurements (Barber et al., 2011), and the effect is considered negligible compared with the overall between‐group differences. Achilles tendon lengths were measured as straight‐line distances, thus neglecting the potential influence of curvature within structures in both groups. However, straight‐line measures of Achilles tendon length have been shown to underestimate length only slightly by ~5 mm (Stosic & Finni, 2011). Therefore, as our significant between‐group differences in tendon length are large (33–40 mm), it is unlikely that this has biased our conclusions. Finally, our sample size of children who ITW may appear small, however this is representative of the small population of children who bilaterally walk in equinus with no known cause, and who have not undergone any recent clinical intervention. Our sample includes children with good variability in age, stature, mass and equinus severity, yet we have still detected statistically significant and clinically meaningful (large ESs) differences between the groups. Nonetheless, further work is required to establish the muscle‐tendon architecture of other muscle groups, and how these change throughout development and in response to treatment.

To conclude, contrary to our hypothesis, children who ITW had longer absolute and normalised muscle and fascicle lengths, and a shorter Achilles tendon length than TD children in all considered joint positions. Therefore, greater consideration of the muscle and tendon properties are required when prescribing current clinical interventions which aim to lengthen the MTU. However, further work is required to assess the implications of the greater muscle length on gait function and muscle force production capabilities in children who ITW.

## AUTHOR CONTRIBUTIONS

CH‐A, TO, CM and VB contributed to conception and design of the research. CH‐A contributed to data acquisition and analysis. All authors contributed to the interpretation of the results. CH‐A drafted the manuscript. All authors edited and revised the manuscript and agreed to its submission for publication.

## Data Availability

The data that support the findings of this study are available from the corresponding author on reasonable request.
